# Roles for the IKK-Related Kinases TBK1 and IKKε in Cancer

**DOI:** 10.3390/cells7090139

**Published:** 2018-09-15

**Authors:** Joel K. Durand, Qing Zhang, Albert S. Baldwin

**Affiliations:** 1 Curriculum in Genetics and Molecular Biology, University of North Carolina at Chapel Hill, Chapel Hill, NC 27599, USA; joel_durand@med.unc.edu; 2 Lineberger Comprehensive Cancer Center, University of North Carolina, Chapel Hill, NC 27599, USA; qing_zhang@med.unc.edu; 3 Department of Pathology, University of North Carolina School of Medicine, Chapel Hill, NC 27599, USA

**Keywords:** TBK1, IKKε, innate immunity, cancer signaling, autophagy, KRAS

## Abstract

While primarily studied for their roles in innate immune response, the IκB kinase (IKK)-related kinases TANK-binding kinase 1 (TBK1) and IKKε also promote the oncogenic phenotype in a variety of cancers. Additionally, several substrates of these kinases control proliferation, autophagy, cell survival, and cancer immune responses. Here we review the involvement of TBK1 and IKKε in controlling different cancers and in regulating responses to cancer immunotherapy.

## 1. Introduction

**Figure 1:**
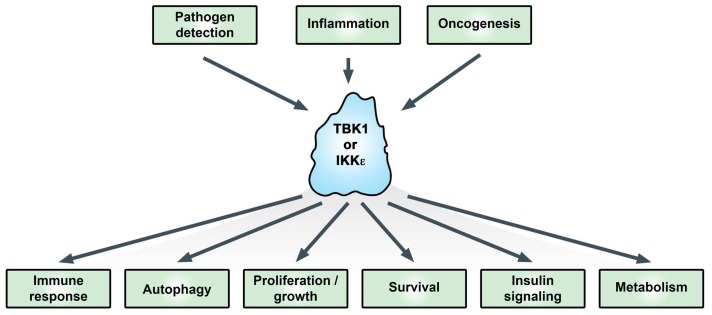
Functional effects of the IκB kinase (IKK)-related kinases. In addition to immune responses, IKKε and TANK-binding kinase 1 (TBK1) are important signaling proteins for critical cellular processes associated with cancer. For more information see text. Adapted from [19].

TANK-binding kinase 1 (TBK1) and the homolog IκB kinase (IKK) epsilon (IKKε, originally IKKi) have been studied extensively in relation to their functions in promoting the type I interferon response. Activation of TBK1 and IKKε promotes interferon regulatory factor (IRF3 and 7) phosphorylation and nuclear translocation, leading to transcriptional upregulation of type I interferons in the innate immune response [1,2]. More recently, these kinases have been linked with adaptive immunity, autophagy, and oncogenesis (Figure 1) [3,4,5]. TBK1 was discovered based on its interaction with TANK [6]. IKKε was identified as an inducible kinase (IKKi) related to the IκB kinases IKKα and IKKβ [7] and as part of a phorbol 12-myristate 13-acetate (PMA)-inducible kinase complex [8]. While TBK1 knockout is embryonically lethal due to liver apoptosis [9], IKKε knockout is viable but exhibits enhanced susceptibility to viral infection [10] and elevated obesity in response to a high-fat diet [11].

**Figure 2:**
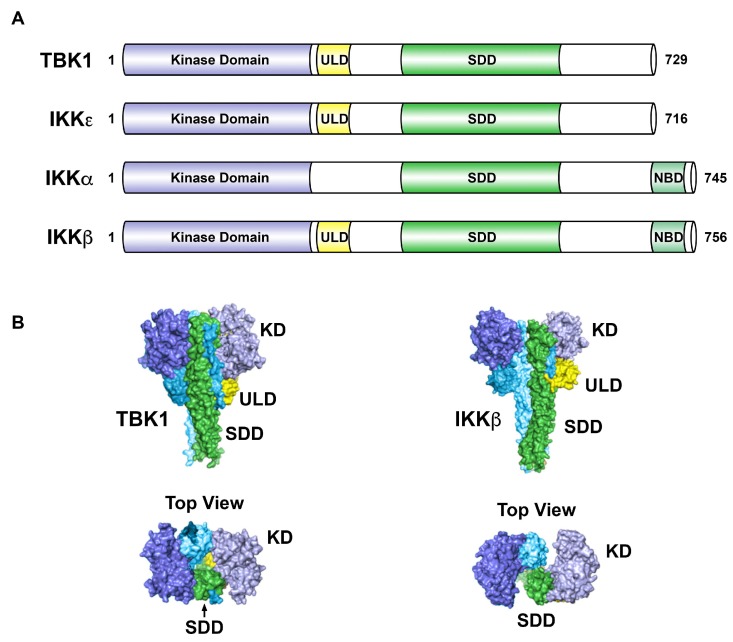
Structural comparison of IKK-related kinases. (**A**). The kinase domain of IKKε shares 27% identity with IKKα and 24% identity with IKKβ. TBK1 shares 49% identity and 65% similarity with IKKε. (**B**). Surface views of TBK1 (left panels) and IKKβ (right panels), with corresponding domains colored in TBK1 and IKKβ. In TBK1, the ULDs bridge between dimer SDDs, but extend away from the opposite SDDs in IKKβ. The kinase domains in the IKKβ dimer are differently oriented and do not form dimer contacts. The IKKβ structure is drawn from Protein Data Bank ID code 3QA8 [28]. The TBK1 structure is drawn from Protein Data Bank ID code 4IM0 [29]. TBK1, TANK-binding kinase 1; IKK, IκB kinase; KD, kinase domain; ULD, ubiquitin-like domain; SDD, scaffold dimerization domain; NBD, NEMO-binding domain. Adapted from [5,29,30].

Downstream of cytokines, toll-like receptor (TLR) signaling, and activation of certain oncoproteins, the canonical IKK complex is activated to phosphorylate IκBα, leading to its proteasome-directed destruction allowing the p50-RelA/p65 dimer to accumulate in the nucleus and drive expression of genes encoding cytokines, anti-apoptotic factors, and other regulatory proteins [12]. The canonical IKK complex is comprised of two catalytic subunits, IKKα and IKKβ, along with the regulatory subunit IKKγ (or NEMO, NF-κB essential modulator). In the non-canonical nuclear factor kappa B (NF-κB) pathway, NF-κB inducing kinase (NIK) induces IKKα to phosphorylate p100/NF-κB2, which leads to p100/NF-κB2 processing into the p52 subunit. The p52 subunit forms a dimeric transcription factor with RelB to drive gene expression [12]. NF-κB signaling is strongly associated with cancer through numerous mechanisms [13,14]. IKKε and TBK1 are referred to as non-canonical IKKs as they have sequence homology with the canonical IKKs, IKKα and IKKβ (Figure 2).

In the innate immune response, TBK1 and IKKε exhibit functional redundancies, although TBK1 appears to be more important than IKKε. In response to pathogen infection, cyclic GMP-AMP synthase (cGAS) binds cytoplasmic, pathogen-derived DNA to generate cyclic GMP-AMP (cGAMP), which binds to stimulator of interferon genes (STING), leading to co-recruitment of TBK1 and IRF3/7 promoting the activity of these transcription factors [2]. The IKK subunit NEMO functions to promote TBK1 and IKKε activation downstream of cytoplasmic DNA signaling, whereby ubiquitinated NEMO recruits IKKβ to facilitate activation of TBK1 or IKKε [15]. Interestingly, the NEMO-related protein optineurin (OPTN) promotes TBK1 activity in immune signaling [16]. In the innate immune response, TBK1 also phosphorylates signal transducer and activator of transcription 6 (STAT6) at Ser407 [17] and STAT3 Ser754 [18] to promote key regulatory aspects of signal transduction and gene expression. IKKε has been shown to phosphorylate STAT1 Ser708 in the antiviral response to drive the transcription of a subset of interferon-responsive genes [10]. To promote an autophagic response involved in pathogen clearance, TBK1 phosphorylates p62/SQSTM1 at Ser403 and OPTN at Ser473 (see below).

In non-cancer diseases, TBK1 has been shown to drive neuroinflammation and autoimmunity [20]. It has been discovered that mutations in TBK1 are associated with several central nervous system (CNS) diseases: amyotrophic lateral sclerosis (ALS), frontotemporal dementia (FTD), normal tension glaucoma (NTG), and childhood herpes simplex encephalitis (HSE) [21]. Gain-of-function mutations in TBK1 underlie NTG, while loss-of-function mutations correlate with ALS, FTG, and HSE. In the case of ALS, evidence suggests that mutant TBK1 leads to aberrant autophagy (see below) and neuroinflammation [22]. IKKε was reported to phosphorylate c-Jun associated with arthritis [23].

Relative to autoimmunity, TBK1 has been shown to promote immune tolerance, at least partly through controlling dendritic cell functions. Work from Sun and colleagues [24] showed that TBK1 functions in dendritic cells to regulate immune tolerance and to suppress autoimmunity. Dendritic cells are the major antigen presenting cells and are required for stimulating the T cell response in an infection. Mechanistically, TBK1 suppresses expression of certain interferon-responsive genes in dendritic cells, which could be one mechanism explaining the immune tolerance effect of TBK1. Interestingly, both TBK1 and IKKε have been shown to inhibit T cell functions [25,26]. In the case of IKKε, evidence is presented that it phosphorylates nuclear factor of activated T cells (NFATc1) to suppress T cell activation [26]. The deubiquitinase CYLD was recently shown to be phosphorylated by IKKε/TBK1 after T-cell receptor (TCR) stimulation [27]. Additionally, TBK1 deletion in dendritic cells enhances response to immunotherapy [24]. By affecting immune tolerance, TBK1 and IKKε could promote tumor progression (see below).

Below we discuss mechanisms whereby TBK1 and IKKε drive an oncogenic phenotype and contribute to responses to immunotherapy.

## 2. Cancers Controlled by TBK1 and/or IKKε

IKKε was reported to be amplified in breast cancer where its expression is important for survival [31]. Further, it was shown that a subset of triple-negative breast cancer requires IKKε for cytokine production and growth/survival [32]. IKKε is implicated in the glioma oncogenic phenotype, where its expression was found to be upregulated in around 50% of gliomas, independent of grade. Cell-based studies indicate that IKKε is involved in promoting glioma cell survival through upregulation of Bcl-2, via an NF-κB-dependent pathway [33]. Interestingly, it was found that IKKε expression is elevated in around 65% of pancreatic ductal adenocarcinomas and is correlated with poorer survival [34]. IKKε expression in ovarian cancer correlates with poor outcome and promotes resistance to several chemotherapies [35]. Hsu et al. [36] found that IKKε exhibits elevated expression in metastatic ovarian cancer, and the loss of IKKε reduces aggressiveness in ovarian xenograft models. IKKε expression has also been shown to correlate with poor outcome and advanced tumor grade in esophageal squamous cancer [37]. IKKε is reported to be important in KRAS-positive pancreatic models, in which oncogenic KRAS drives disease similar to human pancreatic cancer. In this model, IKKε can promote growth and survival via Akt and drive nuclear translocation of the zinc finger protein, glioma-associated oncogene 1 (GLI1) [38]. In prostate cancer cell-based and xenograft models, IKKε promoted proliferation and tumor growth along with interleukin 6 (IL-6) expression in a manner dependent on the nuclear accumulation of the transcription factor C/EBPβ [39]. It was found that epidermal growth factor receptor (EGFR) directly phosphorylates IKKε (Y153, Y159) to promote the oncogenic phenotype of non-small cell lung cancer cells [40].

Studies suggest that TBK1 plays an important role in some KRAS mutant cells by promoting cell survival/proliferation [41]. Additionally, TBK1 has an oncogenic role in melanoma [42] and non-small cell lung cancer [43]. One melanoma study focused on cancer resistant to inhibitors of BRAF, a commonly mutated protein in melanoma, and showed that a subset is sensitive to TBK1/IKKε inhibition (compound II, along with other inhibitors) [42]. In a non-small cell lung cancer (NSCLC) study, a subset of cancer cells exhibited sensitivity to TBK1 inhibition, which was correlated with activation of Akt and mTORC1 (mechanistic target of rapamycin complex 1) signaling [43]. TBK1 and IKKε were shown to promote survival of HTLV-1 (human T-cell leukemia virus type 1) transformed T lymphocytes through the maintenance of STAT3 activity [44]. In breast cancer, TBK1 has been shown to phosphorylate the estrogen receptor (Ser305) to promote its activity and drive resistance to the ER antagonist tamoxifen [45].

## 3. Control of IKKε and TBK1, and Downstream Signaling

The activities of IKKε and TBK1 are regulated by a number of posttranslational modifications. Phosphorylation of TBK1 at Ser172 is known to strongly promote TBK1 activity, and Ma et al. [46] described structural studies supporting a model whereby TBK1 activation involves trans-autophosphorylation (Ser172). Cohen and colleagues have proposed that there is a distinct upstream kinase for TBK1 [47]. Initially, it was found that IKKε (IKKi) is inducible by inflammatory cytokines and lipopolysaccharide (LPS) [7]. Interestingly, IKKε activity is regulated by K63-linked polyubiquitination controlled by the cellular inhibitor of apoptosis proteins cIAP1 and cIAP2, and the E3 ubiquitin-protein ligase, TNF receptor-associated factor 2 (TRAF2) [48]. The authors indicate that this is required for the transforming potential of IKKε. Tu et al. [29] and Larabi et al. [30] reported the structure of TBK1 dimers and the control of activity by K63-linked polyubiquitination, similar to that reported for IKKε [48]. Also, it was reported that TBK1 is sumoylated at K694, near the C-terminus, to promote its antiviral activity [49]. As discussed below, TBK1 is likely to exhibit activation and substrate-specific interactions via regulation controlled by subcellular localization via interaction with distinct adaptor proteins [19].

NF-κB signaling is mediated downstream of several IKKε/TBK1 substrates. For example, it was reported that IKKε phosphorylates TRAF2 on Ser11 and lead to its K63-linked ubiquitination to promote NF-κB activity and mammary cell transformation [50]. Hutti et al. [51] showed that IKKε phosphorylates the tumor suppressor CYLD (Ser418) to suppress its deubiquitinase activity and to promote oncogenesis. IKKε was proposed to phosphorylate and inhibit the transcription factor Foxo3a as an oncogenic mechanism [52]. TBK1 was shown to promote cell survival through its ability to activate NF-κB (controlling Ser536 phosphorylation of RelA), with subsequent upregulation of PAI-2/serpinB2 and transglutaminase 2 [53]. Jin et al. [54] showed that TBK1 can phosphorylate NIK to inhibit noncanonical NF-κB activation, in relation to the immunoglobulin A (IgA) class switch in B cells. Our group previously showed that IKKε controls cancer-associated RelA Ser536 phosphorylation [55]. Harris et al. [56] reported that TBK1/IKKε can phosphorylate the cRel NF-κB subunit to promote nuclear accumulation. While not studied in cancer, it was reported that IKKε can phosphorylate RelA at Ser468 to promote transactivation potential [57].

## 4. Subcellular Localization and Target Specificity

As described above, innate immune signaling activates STING (bound by cGAMP) to recruit both TBK1 and IRF3 to drive phosphorylation of IRF3, such that loss of STING expression blocks this specific activity of TBK1. Thus, STING is a critical adaptor to appropriately direct TBK1 to the biologically relevant substrates. Goncalves et al. [58] analyzed scaffold proteins important for TBK1 and IKKε activity. TBK1 and IKKε are known to interact with three adaptors: TANK, Sintbad and NAP1. This group found that there is a mutually exclusive interaction between the kinases and these adaptors, and that the adaptors are found in distinct subcellular locations. Binding of TBK1 to these adaptors was mapped to the coiled-coil region 2 of the TBK1 C-terminus (Figure 2). In response to viral infection or to cytoplasmic double-stranded DNA, it was found that TBK1 activation was dependent on its interaction with TANK. Specific roles for Sintbad and NAP1 as TBK1 and/or IKKε adaptors need further elucidation. The concept of distinct subcellular localization, due to interactions with critical adaptor proteins, should always be considered relative to regulation of TBK1 or IKKε activity in distinct cell types and under different stimulatory conditions. Helgason et al. [19] have reviewed the concept of subcellular localization in controlling TBK1 activity.

## 5. TBK1 and Mitosis

TBK1 has been reported to directly phosphorylate Akt (see below), an activating kinase of PLK1 [43,59,60]. However, it was reported that TBK1 knockdown decreased viability of A549 lung adenocarcinoma cells, but the authors did not find an effect on Akt activity [61]. Mass spectrometry to analyze changes in phosphoproteins demonstrated that polo-like kinase 1 (PLK1) exhibited reduced phosphorylation (Thr210) upon TBK1 knockdown. Additionally, it was found that TBK1 can phosphorylate PLK1 in vitro. Interestingly, TBK1 phosphorylation (pSer172) was increased during mitosis, and loss of TBK1 blocked mitotic-associated phosphorylation of PLK1. However, Pillai et al. [62] found that PLK1 overexpression was incapable of rescuing TBK1 knockdown cells. This group then showed that TBK1 is associated with the centrosome and identified CEP170 and NuMA, proteins involved in mitosis, as TBK1 substrates (Table 1). Clearly, effects on mitosis should be considered as an underlying growth restrictive mechanism in cancers where TBK1 is inhibited. These studies did not analyze whether there were overlapping, or distinct, effects of IKKε on mitosis.

## 6. Autophagy Regulation by TBK1

Autophagy promotes clearance of damaged cellular proteins and organelles and is a key source of energy and survival for cells under stress [63]. Importantly, autophagy is critical for clearance of pathogens in the innate immune response. Additionally, autophagy has been studied extensively for its involvement in a variety of diseases, including cancer where many (but not all) cancers appear to depend on autophagy for tumor cell growth [64,65,66]. TBK1 has been reported to be involved in autophagy at several levels, consistent with its role in the innate immune response. It was shown that phosphorylation of the autophagy receptor optineurin (Ser177) by TBK1 promotes clearance of intracellular bacteria [67,68]. Furthermore, phosphorylation of p62/SQSTM1 on Ser403 by TBK1 controls autophagy as well as autophagosomal engulfment of mitochondria [69,70]. Interestingly, p62/SQSTM1 has been linked with cancer development and progression [71]. Additionally, Prabakaran et al. [72] reported that the phosphorylation of p62/SQSTM1 on Ser403 by TBK1 limits the innate immune response through degradation of STING. In this role, activation of TBK1 through the STING pathway ultimately feeds back to limit the duration of the innate immune response.

Kimmelman and colleagues have reported that autophagy is important in sustaining pancreatic cancer growth [73,74]. The latter study indicated both cell-autonomous and non-autonomous roles for autophagy in this cancer. Using a model of pancreatitis, it was found that loss of ATG5 to block autophagy, promoted activation of TBK1 in vivo, which led to enhanced T cell-infiltration and programmed death-ligand 1 (PD-L1) upregulation [3]. Upregulated TBK1 activation also led to enhanced expression of IL6, CCL5, and other neutrophil and T cell chemotactic cytokines. Treatment with the TBK1/IKKε/Janus kinase (JAK) inhibitor CYT387 inhibited autophagy and suppressed PD-L1 expression, blocking KRAS-driven pancreatic dysplasia. Thus, TBK1 appears to activate basal autophagy in this model; however, pTBK1 is also degraded by autophagy to prevent excessive TBK1 activity [3]. Others found that autophagy markers LC3 and p62 were elevated in a KRAS-positive pancreatic cancer model where TBK1 was deleted [75]. The interplay between TBK1 and autophagy is relatively clear in pathogenic responses but appears quite complex in cancer.

## 7. Promotion of KRAS-Induced Oncogenesis and Control of Akt

Activating KRAS mutations occur frequently in cancers and are usually drivers of tumor initiation and cancer progression [76]. White and colleagues reported that RalB/Sec5 activated TBK1 to promote cancer cell survival in a study focused on Ras-induced transformation [77]. Interestingly, RalB/Sec5 are involved in the innate response and TBK1 activation [77,78]. Barbie et al. [41] reported that TBK1 knockdown induced cell death in a panel of KRAS-positive cancer cells, with evidence that TBK1 drives pro-survival signaling through an NF-κB pathway involving c-Rel and BCL-XL. Subsequently, the same group showed that TBK1 and IKKε promote the KRAS-driven tumorigenic phenotype through regulation of CCL5 and IL6 [79]. The compound CYT387, a JAK/TBK1/IKKε inhibitor, blocked cytokine signaling and suppressed KRAS-driven lung tumor growth. Muvaffak et al. [80] reported that TBK1 knockdown/inhibition did not affect survival using a panel of KRAS-positive cells, even though in some cells TBK1 inhibition blocked IRF3 phosphorylation. One consideration relative to these studies, is that potential redundancy with IKKε was not considered.

Ou et al. [59] showed that TBK1 promotes Akt activation in cancers but not downstream of insulin, with evidence of direct phosphorylation of Akt. Additionally, Guan and colleagues reported that IKKε and TBK1 can activate Akt by direct phosphorylation on both Thr308 and Ser473 [81]. However, White and colleagues reported that the ability of TBK1 to support Akt activation is context dependent [43]. For example, TBK1 promotes Akt/mTORC1 in response to amino acid addition in starved cells. shRNA kinome screening identified TBK1 as a therapeutic target for human epidermal growth factor receptor 2 (HER2)-positive breast cancer [82]. In this case, TBK1 did not regulate Akt phosphorylation in HER2+ cancer cells. Instead, TBK1 was shown to regulate RelA/p65 phosphorylation, and TBK1 downregulation led to cell-cycle arrest and the upregulation of p16Ink4a. TBK1 inhibition plus lapatanib (an EGFR family inhibitor) strongly blocked xenograft tumor growth. In KRAS-positive cancers, TBK1 involvement in regulating Akt/mTORC1 was found in cells with a mesenchymal phenotype [43]. A recent paper potentially explains these findings, identifying TBK1 activation downstream of endogenous retroviruses that are activated in the mesenchymal state, in association with oncogenic KRAS and other contexts [83]. Based on this, it is likely that conclusions regarding TBK1 sensitivity in KRAS-positive cells, as well as other cancers, will be dependent on factors that relate to the differentiation status of the cells in question.

## 8. TBK1 and IKKε Control of mTORC1 and Metabolism

**Figure 3:**
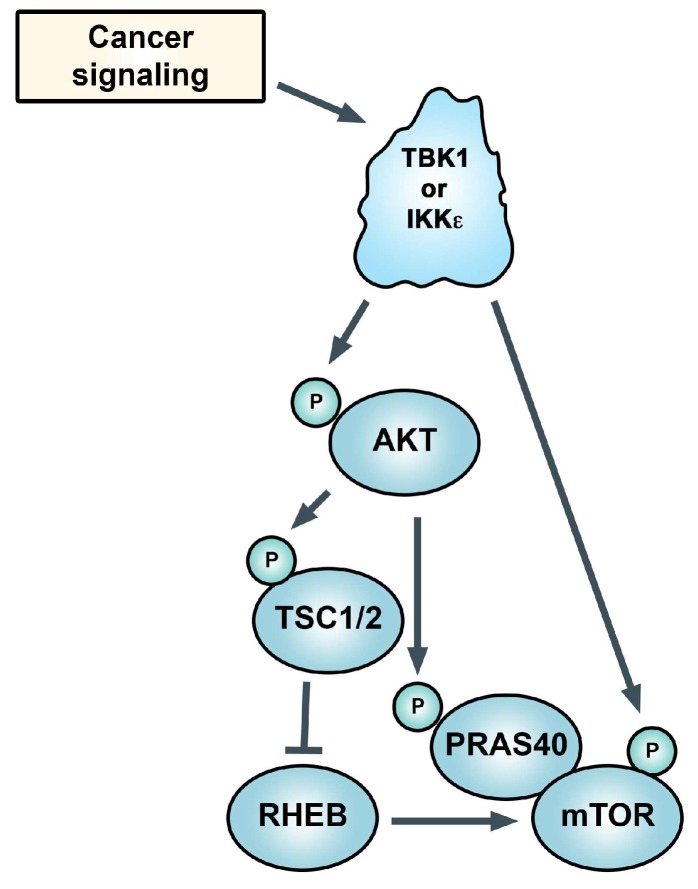
TANK-binding kinase 1 (TBK1) and IκB kinase epsilon (IKKε) control Akt phosphorylation and its activity (in some settings), which drives mechanistic target of rapamycin complex 1 (mTORC1) activity. TBK1 is also reported to directly phosphorylate mTOR. See text.

mTORC1 controls metabolic processes and promotes oncoprotein-induced cell proliferation and survival through phosphorylation of key substrates including factors that promote protein translation [84]. Yu et al. [25] established TBK1 as a regulator of the Akt-mTORC1 signaling axis. mTORC1 is activated downstream of Akt via the ability of Akt to phosphorylate and inactive TSC2 (an inhibitor of mTORC1) and via direct phosphorylation of the mTORC1-associated protein PRAS40 (Figure 3). It was reported recently that TBK1 can directly phosphorylate mTOR, in the mTORC1 complex, at Ser2159 to positively regulate its activity [85]. This mechanism is involved in IRF3 nuclear translocation and IFNβ production. However, Hasan et al. [86] showed that chronic innate immune activation suppresses mTORC1 via TBK1 activation, and Kim et al. [87] showed that overexpression of TBK1 suppressed mTORC1. Consistent with these overall findings, TBK1 was shown to associate with components of the mTORC1 complex [43]. Given that mTORC1 is known to suppress autophagy and TBK1 is known to promote autophagy, one would argue that TBK1 should suppress mTORC1 [88]. It is interesting to speculate that under conditions where TBK1 positively regulates mTORC1 that mTORC1 may not function to suppress autophagy.

It is known that IL-1 activation of mTORC1 regulates Th17 cell survival and effector functions. Gulen et al. [89] found that IKKε phosphorylates Ser21 of GSK3α, (glycogen synthase kinase 3-alpha), blocking the ability of GSK3α to suppress Akt activation. Thus, IKKε has a role in Th17 cell function to promote Akt and mTORC1 activation. IKKε is critical in IL-17-dependent signaling by phosphorylating Act1 (a key interacting protein for the IL-17 receptor) on Ser311 [90]. IKKε also promotes the maintenance of Th17 cells by phosphorylating GSK3α at Ser21 [89]. More studies need to be performed to address the complex interplay between TBK1/IKKε, Akt, and mTORC1.

A hallmark of cancer is that tumor cells maintain rapid growth by switching to glycolysis for a needed supply of energy and macromolecules [91]. Importantly, glucose uptake is elevated in cancer and this appears to be controlled by increased expression of glucose transporters. It was reported that upon activation of RalA, TBK1 phosphorylates the exocyst protein Exo84, which leads to translocation of the GLUT4 glucose transporter to the cell membrane [92]. Thus, TBK1 is involved in insulin-stimulated glucose uptake. Interestingly, TBK1 can phosphorylate the insulin receptor (Ser994) to block the activity of the receptor, potentially leading to insulin-resistance [93]. Other data link TBK1 with regulation of cell metabolism. Zhao et al. [94] have studied the involvement of TBK1 in adipocytes in animals fed a high fat diet, showing that knockout of TBK1 in adipocytes blocked high fat diet-driven obesity. TBK1 is proposed to directly inhibit AMP-activated protein kinase (AMPK) activity via phosphorylation and thereby block respiration and increase energy storage. Interestingly, activation of AMPK under catabolic conditions was found to increase TBK1 activity via direct phosphorylation by ULK1 (AMPK-activated unc-51 like autophagy activating kinase 1). Thus, TBK1 could affect cellular respiration and the switch to the glycolytic pathway. Along a different line, AMPK was recently found to phosphorylate and stabilize the tumor suppressor TET2 (tet methylcytosine dioxygenase 2) [95]. In that study, elevated glucose blocked AMPK activity leading to destabilization of TET2 and reduced 5-hydroxymethylcytosine, related to DNA methylation status. It is interesting to speculate that the ability of TBK1 to negatively regulate AMPK could lead to reduced TET2 and associated epigenetic changes.

IKKε was shown to be overexpressed in around 80% of pancreatic tumors [96]. That study showed that loss of IKKε in pancreatic cancer cell lines reduced cell growth in association with loss of glucose consumption and of the expression of genes associated with glucose metabolism which may be related to the downregulation of c-Myc induced by suppression of IKKε. It was proposed that the ability of IKKε to activate Akt (see above) leads to subsequent Akt-dependent phosphorylation of GSK-3β Ser9 which is inhibitory. GSK-3β phosphorylation of c-Myc is proposed to destabilize c-Myc [97].

## 9. TBK1 and Antitumor Immunity

As described above, TBK1 regulates autoimmunity, at least partly through control of dendritic cell function [98]. While immune tolerance is important for prevention of autoimmune disease, it is also thought to be important for overcoming immune responses against neoplastic lesions. Using both thymoma and melanoma models, it has been shown that specific deletion of TBK1 in dendritic cells leads to reduced tumor growth and increased survival. This is associated with enhanced T cell infiltration into tumors [24]. Barbie and colleagues used organotypic tumor spheroids (which retain lymphoid and myeloid components) to implicate both TBK1 and IKKε in promoting resistance to anti-PD-1 therapy [99]. In a colorectal tumor model, Jenkins et al. [99] showed that PD-L1 inhibition combined with TBK1 inhibition (compound 1) led to a stronger anti-tumor response than either treatment alone. This group argues that the effect of TBK1/IKKε is at the level of T cells, where IL-2 and IFNγ are increased with inhibition, and at the tumor cell level where TBK1/IKKε inhibition leads to decreased C-C motif chemokine ligand 5 (CCL5) and IL-6. Consistent with this overall model, TBK1 was identified (among others) in a CRISPR screen as promoting resistance to immunotherapy in a melanoma tumor model [100].

Mechanisms for TBK1 and IKKε expression/activation in tumors is likely to be varied, and the link with STING is not clear. Findings by Cañadas et al. [83] suggest that TBK1 is activated downstream of retroviruses in mesenchymal subpopulations co-expressing STING (see above). Interestingly, STING expression is often reduced or lost in many cancers, suggesting that STING is not driving TBK1/IKKε activation widely in cancers [101]. STING is detected in human pancreatic ductal adenocarcinomas and in colorectal cancer, but there is a significant loss of STING in advanced disease [101,102]. STING is activated in APCs, as well as stromal T cells, and APCs are known to play a critical role in the effects of STING agonists being tested for antitumor effects [103].

## 10. DNA Damage and Cancer: Is TBK1 Involved?

Cytoplasmic DNA is sensed as a danger signal related to pathogen infection, as described above. cGAS is bound by cytoplasmic DNA to generate cGAMP, which engages STING to recruit TBK1 to phosphorylate IRF3, leading to interferon production. Interestingly, cGAS can be activated by self-derived DNA, including mitochondrial DNA, endogenous retroviral DNAs, and micronuclei generated from DNA damage [104,105]. Importantly, chromosomal instability in cancer which leads to cytoplasmic DNA drives a cGAS–STING pathway to promote metastasis, although a link with TBK1 activation (as measured via phosphorylation of IRF3 and interferon production) was not detected [106]. The authors provided evidence that noncanonical NF-κB activation is associated with these mechanisms and is important for the metastatic potential. Interestingly, it is known that STING activation can lead to non-canonical NF-κB activation in a manner independent of TBK1 [1]. The studies on cancer-associated chromosomal instability and STING signaling need to include methods other than measurement of phosphorylation of IRF-3 in order conclude that TBK1 is not involved.

## 11. Therapeutic Potential and Future Considerations

While TBK1 and IKKε inhibitors have been developed and used in several cell-based studies and animal models, no clinical trials have been initiated related to cancer [20,107,108,109]. Louis et al. [110] showed that TBK1 inhibition with a small molecule inhibitor (WEHI-112) blocked inflammatory arthritis in antibody-dependent models of this disease. A clinical trial was initiated with amlexanox, an inhibitor of IKKε and TBK1, in patients with diabetes and the results showed improved glucose control in the treated patients [111]. One preclinical cancer study involved the use of BX795, an established but not specific TBK1 inhibitor, in blocking oral squamous cell carcinoma xenograft growth [112]. Compound 1, a dual TBK1-IKKε inhibitor (with preference for TBK1) was shown to potentiate anti-PD-L1 therapy in a xenograft model [99]. Eskiocak et al. [42] found that melanomas that are resistant to BRAF/mitogen-activated protein kinase kinase (MEK) inhibitors are sensitive to a TBK1/IKKε inhibitor (compound II). Future cancer studies need to consider potential effects of TBK1/IKKε inhibitors on the likely suppressive effects related to pathogen clearance and on immune system function, such as immune tolerance. An additional concern relates to whether targeting TBK1 or IKKε uniquely would be more (or less) effective for a particular cancer. In this regard, more studies need to be performed to address redundant as well as unique functions for these two related kinases, for a better understanding of the biology associated with TBK1 and IKKε and also to consider potential adverse effects of drug intervention. Proteolysis-targeting chimera (PROTAC) has emerged as a technology that can be used to target an “undruggable” protein for degradation. PROTACs contain one moiety that binds an E3 ligase linked with another moiety that binds to the target protein. The induced proximity results in ubiquitination and subsequent degradation of the target. Recently, Crews and colleagues demonstrated that a PROTAC directed to TBK1 can specifically degrade TBK1 in cells while not affecting the IKKε [113]. Thus, utilization of a TBK1 PROTAC could functionally dissect roles of TBK1 from those of IKKε, and also may prove therapeutically beneficial.

## Figures and Tables

**Table 1:** Substrates of IKK-related kinases. The IKK-related kinases regulate biological processes through several different substrates. Act1, NF-κB activator 1; cRel, proto-oncogene; IRF3, interferon regulatory factor 3; IRF7, interferon regulatory factor 7; p65/RelA, NF-κB p65/RelA subunit; PELI1, Pellino-1; STAT6, signal transducer and activator of transcription 6; STING, stimulator of IFN genes; TANK, TRAF-associated NF-κB activator; TBK1, Tank-binding kinase 1; XIAP, X-linked inhibitor of apoptosis protein; OPTN, Optineurin; p62, Sequestosome-1; Akt, Akt-1; CEP170, centrosomal protein of 170 kDa; mTOR, mechanistic target of rapamycin; NuMA, nuclear mitotic apparatus protein; Sec5, Exocyst complex component 2; IR, insulin receptor. For more information see text and [19].

Biological Process	Kinase	Substrate	Protein Function
Immune response/inflammation	TBK1	Act1	E3 ubiquitin ligase
		cRel	transcription factor
		IRF3	transcription factor
		IRF7	transcription factor
		NFATc1	transcription factor
		RelA/p65	transcription factor
		PELI1	E3 ubiquitin ligase
		STAT3	transcription factor
		STAT6	transcription factor
		STING	receptor/adaptor
		TANK	adaptor
		TRAF2	E3 ubiquitin ligase
		XIAP	E3 ubiquitin ligase
	IKKε	c-Jun	transcription factor
Autophagy	TBK1	OPTN	autophagy receptor
		p62	autophagy receptor
Proliferation/growth	TBK1	Akt	kinase
		CEP170	centrosome associated protein
		CYLD	deubiquitinase
		mTOR	kinase
		NuMA	centrosome associated protein
		PLK	centrosome associated protein
		Sec5	exocyst component
Insulin signaling	TBK1	IR	receptor kinase
